# The vasa vasorum of the large pulmonary vessels are involved in COVID-19

**DOI:** 10.4322/acr.2021.304

**Published:** 2021-08-20

**Authors:** Hubert Daisley, Arlene Rampersad, Martina Daisley, Amit Ramdin, Oneka Acco, Farhaana Narinesingh, Ornella Humphrey, Errol James

**Affiliations:** 1 San Fernando General Hospital, Department of Pathology, San Fernando, Trinidad and Tobago; 2 Scarborough General Hospital, Department of Accident and Emergency, Tobago, West Indies; 3 University of the West Indies, Trinidad, West Indies; 4 Beacon Plastic and Cosmetic Surgery Clinic Woodbrook Port- of Spain, Trinidad and Tobago, West Indies

**Keywords:** Blood Vessels, Coronavirus Infection, Vasa Vasorum

DEAR EDITOR

In our manuscript “COVID-19: a closer look at the pathology in two autopsied cases. Is the pericyte at the center of the pathological process in COVID-19?” ;^1^ published in Volume 11, 2021 issue of Autopsy Case Reports; Figures 3B, 4A, 4B, and 4D show thrombosis within the walls of large pulmonary vessels. These locations of thrombosis may represent the location of the vasa vasorum of these pulmonary vessels. Figures 3B and 4A may represent thrombus within vasorum externa, whilst figures 4B and 4D represent thrombus within vasorum interna.^2^

The significance of these sites means that both, the alveolar microcirculation and the vasa vasorum of the large pulmonary vessels are involved in the thrombotic processes in COVID-19. The latter thrombotic site would further implicate COVID-19 as a disease of the microcirculation. Boyle and Haverich^3^ postulated that “the involvement of the large vessels during coronavirus disease 19 (COVID-19) in both children and adults is likely due to dysfunction of their vasa vasorum and SARS-COV-2 induced microthrombosis of vasa vasorum would lead to hypoxic conditions in the adventitia.”

As the world is grappling with the SARS-COV-2 pandemic, it is essential to understand the pathogenesis of COVID-19 to formulate therapeutic options. It may be that the lung is a secondary partner in COVID-19 and the pathogenesis of this disease begins in the nasopharynx,^4^ where there is an abundance of ACE-2 receptor sites. SARS-COV-2 interaction at the port of entry in the nasopharynx produces chemokines/cytokines,^3^^,^ 5 which circulates in the systemic circulation, causing endotheliopathy and subsequent thrombotic microangiopathy^6^ being the ultimate result.
